# A Novel Blockchain and Bi-Linear Polynomial-Based QCP-ABE Framework for Privacy and Security over the Complex Cloud Data

**DOI:** 10.3390/s21217300

**Published:** 2021-11-02

**Authors:** Kranthi Kumar Singamaneni, Kadiyala Ramana, Gaurav Dhiman, Saurabh Singh, Byungun Yoon

**Affiliations:** 1 Department of Computer Science and Engineering, GITAM Institute of Technology, GITAM Deemed to be University, Visakhapatnam 530045, India; kkranthicse@gmail.com; 2 Department of Artificial Intelligence & Data Science, Annamacharya Institute of Technology and Sciences, Rajampet 516115, India; ramana.it01@gmail.com; 3 Department of Computer Science, Government Bikram College of Commerce, Patiala 147001, India; gdhiman0001@gmail.com; 4 Department of Industrial and System Engineering, Dongguk University, Seoul 04620, Korea; postman3@dongguk.edu

**Keywords:** cloud platform, ciphertext policy-based attribute-based encryption, blockchain, hashing, information security, non-polynomial chaotic mapping, quantum key distribution

## Abstract

As a result of the limited resources available in IoT local devices, the large scale cloud consumer’s data that are produced by IoT related machines are contracted out to the cloud. Cloud computing is unreliable, using it can compromise user privacy, and data may be leaked. Because cloud-data and grid infrastructure are both growing exponentially, there is an urgent need to explore computational sources and cloud large-data protection. Numerous cloud service categories are assimilated into numerous fields, such as defense systems and pharmaceutical databases, to compute information space and allocation of resources. Attribute Based Encryption (ABE) is a sophisticated approach which can permit employees to specify a higher level of security for data stored in cloud storage facilities. Numerous obsolete ABE techniques are practical when applied to small data sets to generate cryptograms with restricted computational properties; their properties are used to generate the key, encrypt it, and decrypt it. To address the current concerns, a dynamic non-linear polynomial chaotic quantum hash technique on top of secure block chain model can be used for enhancing cloud data security while maintaining user privacy. In the proposed method, customer attributes are guaranteed by using a dynamic non- polynomial chaotic map function for the key initialization, encryption, and decryption. In the proposed model, both organized and unorganized massive clinical data are considered to be inputs for reliable corroboration and encoding. Compared to existing models, the real-time simulation results demonstrate that the stated standard is more precise than 90% in terms of bit change and more precise than 95% in terms of dynamic key generation, encipherment, and decipherment time.

## 1. Introduction

The various types of IoT applications include smart cities, smart health care, and smart transportation applications [1,2]. The critical entities in an IoT device include the device itself, a sensor, and an actuator. Sensor-equipped IoT devices collect data from their surroundings and transmit it via the Internet to a storage device. Analyses of the data are then conducted to determine the best course of action or share data with remote host machines.

**Figure 1:**
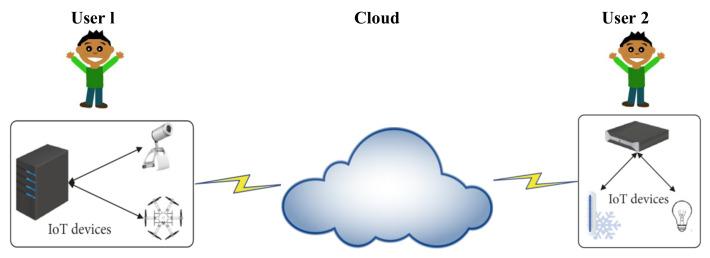
Cloud-Based IoT data sharing.

The data are not stored locally because the local device has limited resources, such as insufficient memory for data storage and insufficient computation capacity for data analysis and action. This will result in the incorporation of cloud computing into the Internet of Things, with cloud computing providing services to IoT devices such as data storage and processing, as exemplified in Figure 1.

As depicted in Figure 1, User 1 collects different states of information from different locations via sensors and stores it in the cloud; these data can then be shared with remote host User 2 via IoT Device 2, which is part of a more cost-effective data-sharing method between communicating entities. However, the issue with this is that the cloud is untrustworthy, as there might be a chance of cloud consumer’s sensitive data leakage. Numerous policies based on cryptographic tools are available to restrict user access and safeguard user privacy [3,4]; however, on rare occasions, an insider at the cloud end can delete these policies, thus jeopardizing the user’s privacy.

To address this issue securely, cloud computing and blockchain technology are combined to store and share IoT data. Blockchain technology is a distributed ledger [5] with numerous applications, including IoT [6,7,8,9], e-currency [10,11], information loading [12,13], and data lineage [14].

We propose a fusion of blockchain and attribute-based cryptosystems [15,16] to address two critical cloud-related issues: preserving user privacy and sharing data in a manner that involves complete user control. This allows the data owner to choose whom to share their information without jeopardizing their data or privacy. This proposal includes the following:

- Data collected from IoT devices are converted to a ciphered text, then combined with an attribute encryption mechanism [17,18], which is then shared in small chunks. The key is then converted to ciphered text. Access policies are applied to the ciphered key using the QHCP-ABE framework for everything from determining who will become the temporary owner of the ciphered key to decrypting the cipher text.
- The smart contract provides scalability by storing access control policies in tables; users requesting to share data will interact with smart contracts via transactions.

Over the fast evolution and growth of the cloud centralized computing and WWW centric operational strategy, there are large volumes of user’s information handling and distribution of resources over the cloud environments [6,7,8,9,13,14,15,16,19]. The external cloud resource manager wants to provide authorized access structure/policy and privacy towards the cloud users. Correspondingly, it is greatly recommended that the large sized apps are enabled one to one, one to multicast, and one to broadcast services to lessen the time and space required for users’ data encipherment and decipherment as in traditional mathematical encryption techniques, since the cloud apps like Google drives and Dropbox, cloud structures like Amazon’s EC2, and platforms like Amazons’ Simple Storage Services (S3), Window’s Azure have dissimilar structures and diverse facilities [20]. In these types of infrastructures and applications, when the cloud user’s data increase more and more in large volumes day by day, extreme confidentiality is important to maintain those data alongside un-approved external apps, software, and manipulators, specifically in the case of public nets and medicinal accounts. Security along with privacy are the key issues in the cloud environment [2,21,22]. The existing old-fashioned cryptographic structure constructed over the public key infra-structure (PKI) may achieve users’ info safety, privacy, and non-repudiation but it is associated with many problems, concerns, and restrictions. In the process of data encipherment, the external cloud authority wants to acquire the official persons’ public keys, i.e., transfer the ciphered text to the users in one to one fashion that leads to bandwidth misutilization or wastage along with more computational time and network overhead. To address these key issues in place of traditional mathematical cryptographic models we replaced advanced Attribute Based Cryptographic schemes which encipher the cloud data only once w.r.t individual user which could be more beneficial to end users. In modern Attribute Based Encipherment (ABE), all users have their individual group of certified attribute subsets, rules/policies, and a secret key [3,4,21,22]. Numerous ABE structures are anticipated on different user’s data but when the volume of user attributes and data increase those models fail to maintain proper access policies to access the user’s confidential data. In the fundamental ABE process, secret and private keys along with ciphered text are also deciphered by using the access policies [3,4]. The end user can only decipher ciphered text contingent upon the corresponding user attribute set fulfilling the access control structure.

ABE is a public key cryptosystem which performs the decipherment of a cipher-text by any person and fulfils the designed access policy based on users’ personal attributes. AB Encipherment scheme is part of the Identity Based Encipherment (IBE) base approach [3,4,21,22]. The idea of ABE is enhanced to integrate sophisticated and effective access control over the user’s confidential data. Through implementing additional broad level access strategies, ABE structures attract the academic world as well as industrial users since these approaches allow the unauthorized users to gain admittance to legitimate users’ personal information deprived of the central admittance regulate arrangement. Above and beyond subsidiary admittance rheostat claims, ABE also supports appliance additional academia and industry attention-grabbing claims like auditing log based encipherment and is directed towards multicast encipherment [4], unlike traditional mathematical encipherment models. In broad ABE cryptosystem two primary and significant schemes have been coined: Key Policy Attribute Based Encryption (KP-ABE) and Cipher text Policy Attribute Based Encryption (CP-ABE). In the past few years CPABE scheme has already been developed, and extensive research has been undertaken on that scheme and it has been instigated in many academic and industrial applications for users’ cloud data security and privacy [2,3,4,21,22]. In this scheme the access policy is assimilated over the user’s ciphered text, and a personal decipherment key is produced over the collection of users’ subgroup attributes. Whatever the attribute held by specific users, only that attributes’ combination should gratify the designed admittance rule/access policy, that a specific user only shall decipher the ciphered text which was enciphered under the designed rule/policy. Aimed at the design of the access structure/policy w.r.t CPABE models that specific access policy/structure should be well-known prior to the encipherment along with the user’s subset of attributes based personal/secret keys. Conversely, KPABE [5,7,21] permits the users’ block of data can be enciphered with the help of users’ personal attributes which act as public-keys. In case of private/secret key generation this scheme uses a certain access policy/structure well-defined above the subset of user’s personal attributes or characteristic based values like bio-metric values, passwords, pins, etc. (For a clear exemplification of the work flow see Figure 1). Due its dynamic and easy implementation, CPABE has been incorporated as a fundamental public key encipherment approach for all types of applications. On the other hand, KPABE has failed to access structure design on users’ data, which is one of the major drawbacks of KPABE.

**Figure 2:**
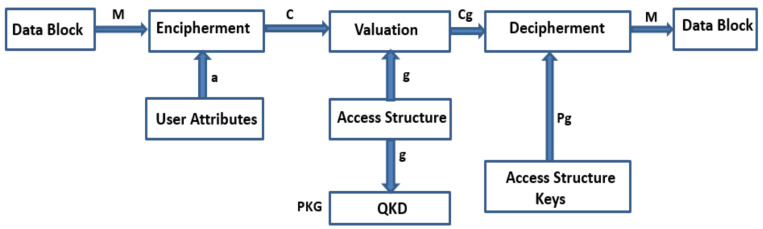
Exemplification of the QHCPABE scheme work flow.

It is worth noticing the analysis of QKD practice in the noise-free channel as a part of base experiment level [17,23,24,25]. Furthermore, for the future reliable and confidential web communication by multiple cloud users, the practical experimental base model is mandatory. Furthermore, we tried the same approach in a noisy channel with the help of QKD model as shown in Figure 2. Due to this unrestricted privacy and confidentiality of quantum cryptographic approach, it is more apt for web applications and cloud computing as forever-growing defies problems which are inexorable in the upcoming period. QKD integrates the discrete session level dynamic secret key generation over numerous cloud users’ over the one-to-one quantum link. QKD is effusively cast-off at abundant cloud consumers’ secret generic procedures to achieve security and privacy [26,27,28]. QKD is derivative of quantum physical science aimed at generation of a secret key that leads to flawless persistence, in every single situation QKD cast-off over many different apps. Safety and privacy should be achieved through their elementary quantum physical particles called photons performance which are reliable, stable, and imperceptible. The prime advantage of integrating QKD with CPABE structure is that this unification provides the advanced confidentiality and privacy for the individual cloud users’ private data.

**Figure 3:**
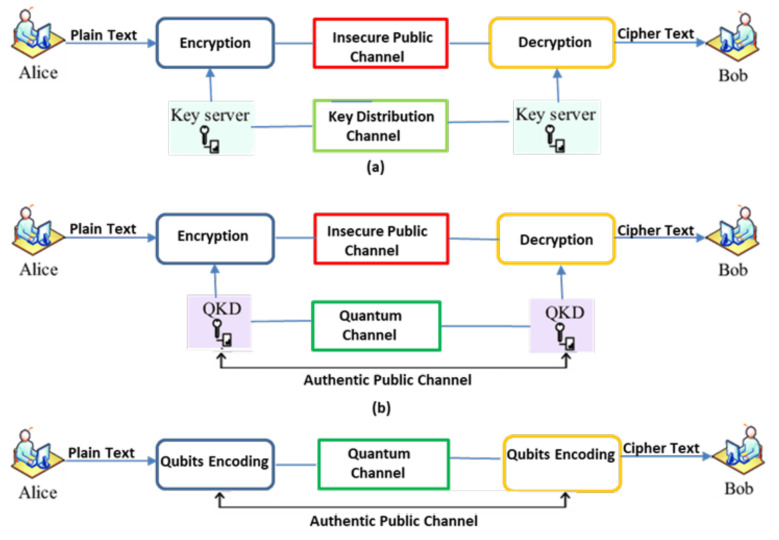
Traditional and Conventional QKD in Cryptographic Representation.

**Figure 4:**
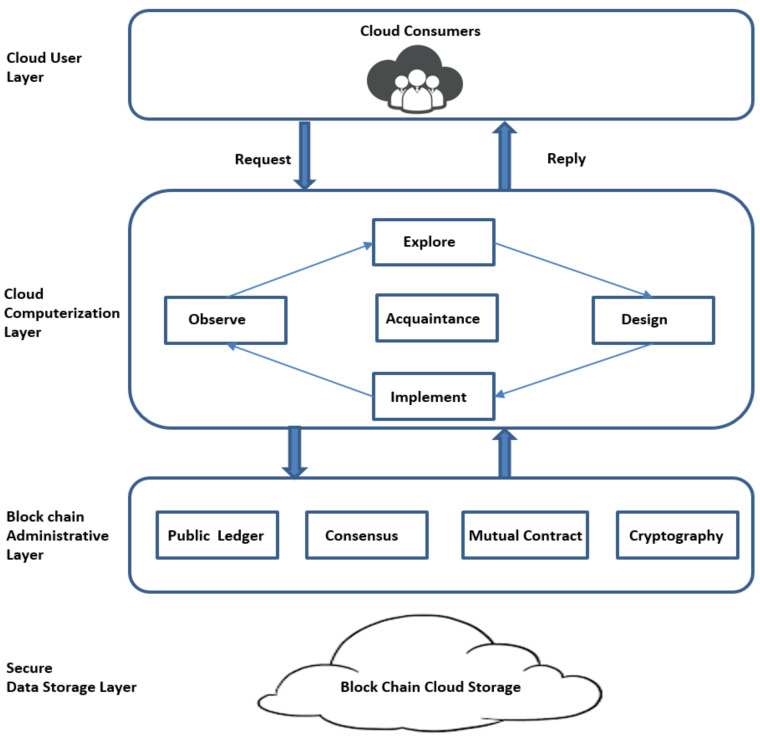
Secure Block chain QCPABE based Cloud Framework.

The distinctive QKD approach is shown in Figure 3. Individual pairs of QKD are linked by a quantum channel. The quantum channel is linked to classical open network channel [29]. In this QKD approach, Sender (Alice) and Receiver (Bob) communicate their cubits via the secure quantum communication link and actual information is communicated with a classical cryptographic approach by means of open communication link [30]. To retrieve the cloud user data, block chain is also an effective technique [31,32,33,34,35]. Automated electrical user’s personal clinical database is the present operational patient monitory facility contributing the main portion of preserving and monitoring the user’s information, which was the key problem in w.r.t confidential patient’s personal files breach [26]. To observe and access the sick person’s personal data or clinical medical reports/records we use block chain related approaches with ledger feature. Block chain provides additional advantages like confidentiality, uprightness, and authentication along with privacy, ease of real time medical and clinical data accessing [18] and other user application oriented data, and administration [36,37,38,39]. Consequently, the key goal of the present contribution is to facilitate strict data privacy and security framework for cloud users’ data by combining the block chain technique with quantum base ciphered text attribute encryption technique for additional security and to avoid man in the middle attacks [40,41,42]. Figure 4 shows how the model provides the security to cloud data.

In this article, a Novel Block Chain Non-linear Polynomial Quantum Ciphered text Policy Attribute Base Encryption model is offered which can be implemented with the help of chaotic randomized non-linear polynomial curves for massive volumes of users confidential cloud data like patient medical records, bank transactions, etc. The planned system is well structured and it can handle large cloud data volumes and also produce an optimum resolution to obtain control over the framework through the different layers as shown in Figure 4.

The projected prototypical framework necessitates a reduced amount of computational time-band and smaller amount of network overhead to perform efficient user’s confidential data encryption, decryption, and key production as compared to traditional models. Moreover, the outmoded representations are not successful in accessing structure rules/policies revocation and policy structures dynamic updating as per necessity. The projected design successfully implements the revocation process and dynamically updates access policy structures by way of less processing overhead parallel. This model comparatively needs lower processing time for block chain based cryptographic process, chaotic dynamic key production with the help of QKD approach, and the QCPABE set of rules. The considerable objects of the proposed model are mentioned here:

1. This one reassures large volumes of cloud consumer confidential data which may be structured and unstructured or both.
2. This one successfully moderates the cryptographic process and key generation time over the large volumes of personal cloud data.
3. This one successfully works on structured, semi structured, unstructured, and hybrid patient clinical records like doc, xls,.pdf, images, decom images, x-rays, etc. and diversified image representations.

## 2. Related Work

Chen, R. et al. [1] examined the personal information by using their biometric values and their confidential data preservation with the chaotic enciphering approaches. Overall the existing approaches are integrated with the traditional randomized enciphering process with the aid of Bernoulli’s logistics basis. This research acknowledged plenty of workers’ sensitive information privacy issues as well as previous issues with bio-metric users’ information apps, and the authors of that study reconnoitered and inspected the previous recognized cryptographic varieties along with demerits of the approaches. Additionally, they mentioned the issues of biometric sensitive information. To overcome those existing problems a developed enciphering process is coined that can be combined with a three dimensional Bernoulli-Logistic family of curves approach. A thorough investigational assessment was accomplished, and the stemmed conclusions demonstrate that the described practice indicates more improved influence in the case of co-relation dissemination and histogram. That study additionally shows that the inefficacy and unreliability of the present approach are less than that integrated with Bernoulli-Logistic family of curves. The approach described above ensures extensive security and flawlessly retains the hiddenness of the encipherment. The outcomes of relative propagation of cloud consumer’s information are more intermingled by dissemination and fabrication. The stochastic qualities of the histogram prove that the enciphering process is improved. Accordingly, it becomes more complicated to break down together the original user message system and the ciphered message system. Similarly, this methodology is very broadly applied in the electronic bio-metric users’ information network [3,4,6]. During the period of PKE, two individual keys are used to encipher mentation and non-scrambling commotion. Both allocated keys are accessible and one more key is used for exclusive personal purpose. Operators in the public key are openly accessible, whereas the personal key is only accessible to the envisioned user. All users’ plaintext is enciphered by the envisioned acceptors’ publicly available key, and the procedure of decipher mentation is completed after the envisioned cloud consumer secret key. This method proposed a purpose to the enormous overheads associated with key administration/administrative; henceforth, it is cost ineffective for cloud computation. Policy based ABE [10,21,22] model addresses the base postulated issues. Moreover, the employers’ characteristic based attribute sets should fulfil the challenging control structure polices, lone the worker only eligible for decipher mentation [7]. This contrived approach is exceptional to wide-open key crypto graphical techniques, stemming from its epicenter functioning rate all over the period compensated for in critical monitoring. In similar scenarios a certain clandestine key of a user is threatened, so only the information of that exact operator might be unscrambled by contemplation of user attributes. Homomorphic hash encipher mentation would be definite for the administration of cloud consumers information for their security and privacy [17]. This is assumed to be an acute practice in the cloud. This approach authenticates the cloud consumer’s sensitive information privacy in determining the fortification problem of resource reservation over the cloud user whether he is an authenticated user or not. Users are expert at making use of resources offered by cloud continuously by the Internet. Thus, the system deploys the inclination cloud features. Abrading and termination pay might be contemplated as dual different practices can directly expand the cloud systems’ user-friendliness and flexibility. In addition, some more block-chain and cloud related work can be read and referred to the references [20,31,32,33,34,35,36,37,38,39,43,44]. The CPABE practice leads to several key problems when installed at cloud consumer’s information interchange policy construction. The secret keys of cloud users are created through the Key Production Center (KPC) though the Master Secret Key based attributes put off by cloud consumers. The suggested pseudo code needs fewer efforts to deposit public key certificates (PKCs) in contrast to whole conventional PKIs’ [1,2]. The approach mentioned above fails to resolve key distribution issues by means of the KPCs which can decode every ciphered text chosen to all exclusive clients using the attributes of the keys’ creation. The indicated issue impinges on users’ information privacy limits of personal information allocating schemes. Another major disadvantage is revocation of key which is already considered as a known problem [8,9,15,16]. In the ABE-Technique, the main problem in the procedure of key revocation/cancelation is the revision of every new characteristic/attribute of both existing and new users. Each user’s characteristic is used by more than the single client, and numerous clients might transmute the acquaintance-based attributes or otherwise alter specific secret keys. This practice of revocation is essential to preserving users’ sensitive information security and users’ information privacy. Every client from the cluster is influenced by one specific attribute or the other.

### Traditonal Cryptographic Techniques Based on QKD

**Figure 5:**
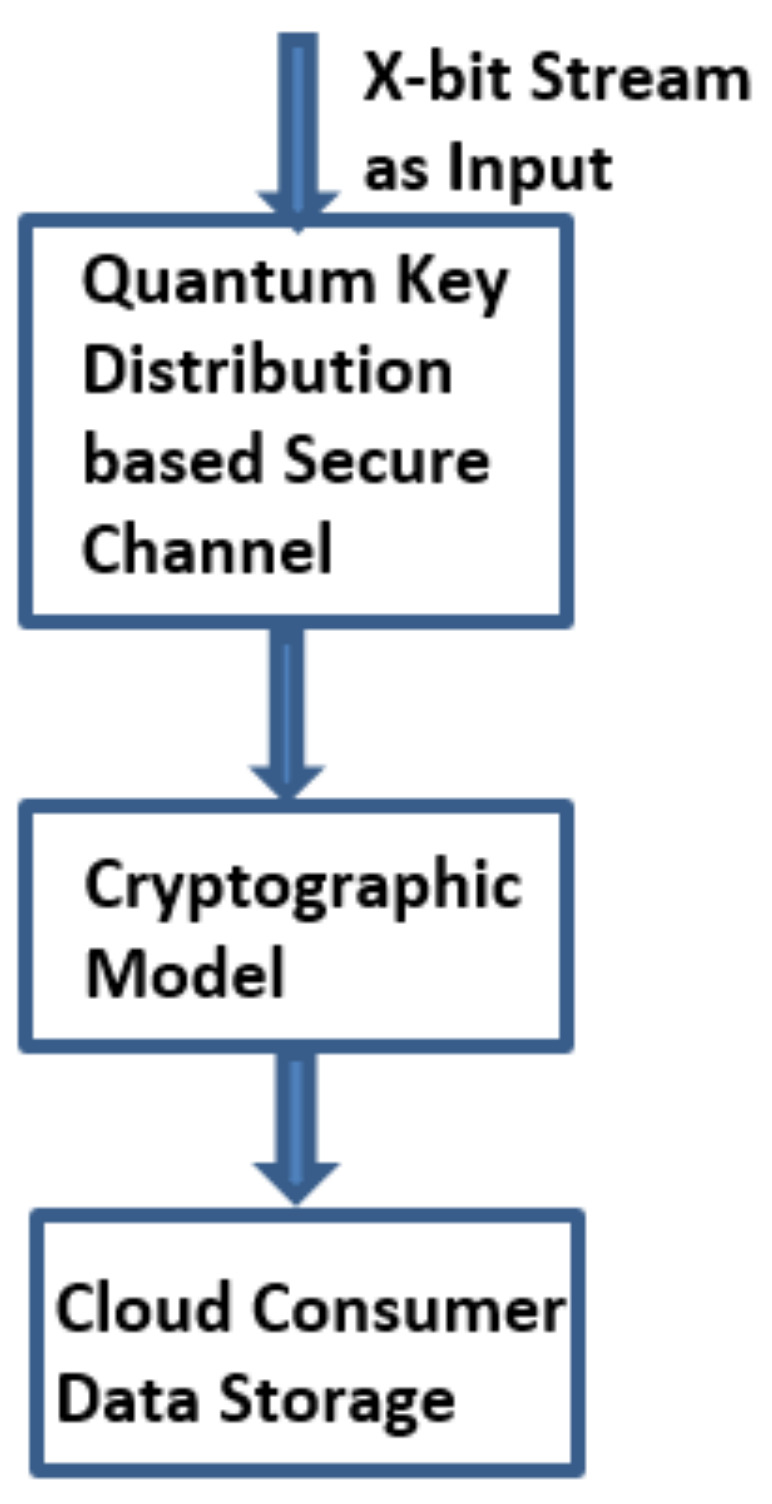
QKDs Cryptographic model.

The main use of QKD is to generate a key with help of cubits which is used for the transmission of private data among sources and destination by using quantum signals without aware of what is the private key value. The QKD process is as shown in Figure 5. Here BB84 protocol for QKD is used, while smearing the crypto graphical approach to offer safety to cloud consumer’s personal information exchange [29,30,44,45,46,47,48].

Tseng F.K. et al. [2] developed an improved biometric base arrangement to protect medicinal electronic health information over the cloud [3,10]. Implementing private electronic health checking applications has resolved many encounters in the healthcare domain, resulting in pointedly additional conjoint welfares for ill people and healthcare professionals. To achieve this goal, HMA techniques must continuously monitor a patient’s history and condition. This is typically accomplished using sensor devices that collect information about sick people as well as diagnostic reports attached to the corpses of sick people. These are considered to be highly confidential and sensitive. As a result, cloud servers must maintain the privacy and security of stored personal and sensitive information about sick people. The purpose of this research paper is to design and advance a protective security structure specifically for cloud centric HMA patient files. This structure has the following advantages.

1. Thwarts unauthorized admittance access after unintended operators or invaders.
2. Whole patient related information is secured by means of the biometric validation scheme.

Yang Y. et al. [21] offered an encipher mentation identifying technique to improve upon the biometric authentication process. The presented research paper designed a novel technique where biometric photographs are gathered with the help of the visual encipher mentation process. This type of techniques is characteristically positioned on the theoretical based assumptions of Compacted Sensing (CS) and Double Random Period Encipherment (DRPE). Furthermore, encoded detecting DRPE is also connected with the Digital Holographic method (DHM). Many studies are conducted at the valuation state. Enciphered bio-metric pictures are taken with the help of thump print pictures and thumb vein pictures. Repair might be accomplished exactly with the gathered encoded photographs’ support.

**Figure 6:**
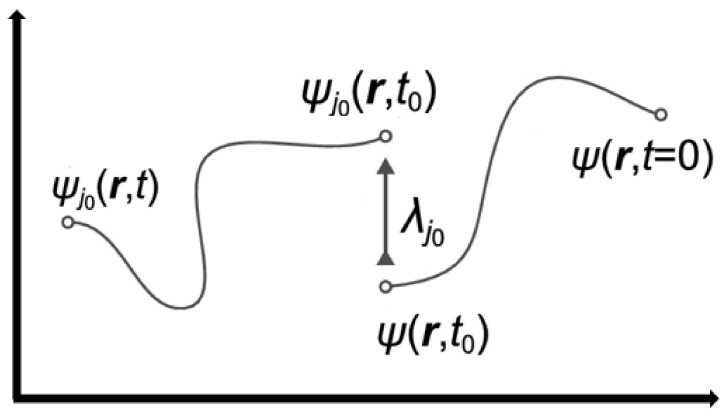
Non-Linear Quantum Chaotic Randomization.

Song X. et al. [22] presented a novel, refined biometric encipher mentation [3,4,11]. In The presented paper, authors castoff a slant authentication technique. The variant tokens are produced with the registration procedure that is typically reserved on biometric database. All the secure tokens are not completely equated along with all newly provided biometric tokens. Such things are considered to be an important facility towards biometric approaches. PIN/password authentication methods typically contain the symmetrical authentication technique. Suppose the required PIN/password may change by chance, apart from the initially selected one. In that case, the authentication action should fail [11]. Due to that, cloud consumers’ trustworthiness should be unauthenticated. In the traditional mathematical research, the utilities in which i/p failed to generate o/p that means anywhere nearby the scope/scale for the given i/p is called chaotic. On behalf of this, Figure 6 shows the bilinear chaotic randomization [5,49].

The quantum state as shown above signifies the part of computing the size of the key which breakdown into an arbitrarily/randomly designated function of this evident, ψj_0_ and the extent outcome is a chaotic random position w.r.t Eigen-value λj0 (any prime number). The outcome is absolutely arbitrary/random and the consequence dimension devastated the previous state of Ψ(r, t) so this is considered the primary condition ψ (r, t = 0) at the time of initiation. None of this progression sequence evokes the past form/state and at every level it arises by way of totally arbitrarily designated function of the noticeable ψj_0_(r, t0).

## 3. Proposed Model

**Figure 7:**
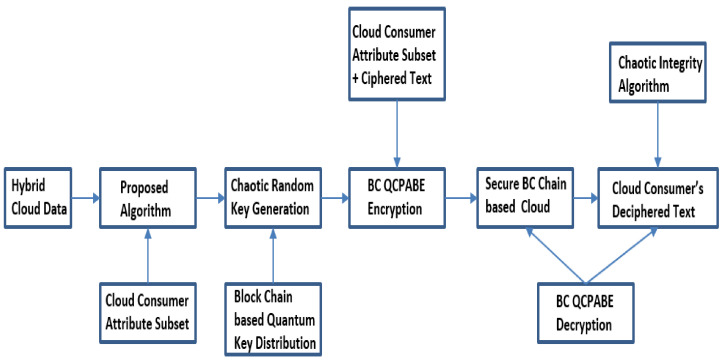
Block Diagram of the Proposed Model.

Proposed block chain and bi-linear polynomial based QCP-ABE need communiqué passages like a quantum network communication link and a normal network communication link. The source user and destination user together need chaotic randomized producers after the bi-linear cyclical cluster and a pair of rudimentary and polarizing cubits [46,47,48]. We implemented this model on the basis of BB84 QKD to avoid the substantial Q link being confronted throughout communiqué from the M-I-M attacks. The rudimentary stages involved in the projected model are presented in Figure 7.

**Figure 8:**
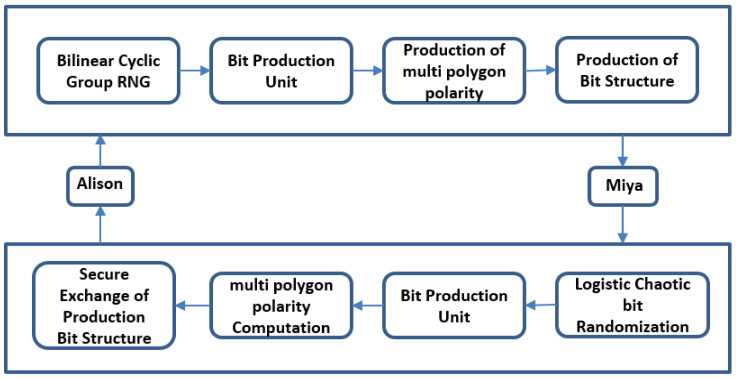
Proposed polygon random polarization-based CBCQKD.

The offered prototypical framework has three methodologies: consumer integrity metric computation, QKRD, and encipher, decipher process of cloud users’ private data. In the initial process the input accepts the cloud consumer attribute values to compute user integrity, and then each user’s value can forward to the secure block chain based quantum key production after implement encipher and decipher algorithms. The next step consists in chaotic integrity metric based quantum key production process in cloud consumer attributes, control structures/policies, and individual session key generation based on CP-ABE [3,15,21,22,25]. The last step consists in the computed user integrity metrics and chaotic quantum key used in the first step of set-up, random key creation, and encipher, decipher processes. The quantum, public, private, and master keys are used on cloud consumer input attributes/characteristics. The ciphered text can only decipher by these attributes/characteristics, and the control structure policy accessed decision tree set in cloud consumer’s ciphered message is represented in Figure 8.

### 3.1. Novel Blockchain and Bi-Lienear Polynomial Based Quantum Ciphered Policy Attribute-Based Encipherment Algorithm (NB-BPQCP-ABE)

A bi-linear map is a function B:X ∗ *Y* → *Z*: ∀ y ∈ Y, the map B_y_ then x → B(x, y) is a bi-linear map from X to Z, and ∀ x ∈ X, the map B_y_: y → B(x, y) is a bilinear map from Y → Z. X, Y, Z are three vector spaces upon the same field F. The bilinear productive cyclical set with ‘o’ has a group order *Order*(*o*) ≤ *Order*(*Z*(*o*^2^, ∗)). Our novel projected pseudo code, a bi-linear chaotic arbitrary polynomial curve, is used for the enhancement of the privacy metrics/input for the session wise key making progress. The rudimentary iterative co-relation of bi-linear randomized polynomial value can be articulated by way of

Z_n+1_ = K Z_n_ (1 − Z_n−3_) (1)

where K is a constant and Z_0_ is the initialization term is computed by the above relation

Z_0_ = 1 (2)

Z_1_ = K Z (1 + K^3^) (3)

Finally, $\frac{\mathrm{dSz}}{\mathrm{dn}}$ indicates a set of bi-linear randomized graphs with exponent entitlements. Here K is a chaotic privacy check metric which can take from the range of Z(m, *) to *Z*(m^n^, ∗). Using the above method can get the different set of bi-linear randomized polynomial graphs along with a wide range of co-factors through determining the recurrence equivalence.

**Inputs:** Start to prepare input cloud user attribute based parameters, message block size BLK_M, Over-all computational serial iterations CS_I, block-bytes, Cyclical confusion, diffusion set CD_S, cloud consumer private data load CP_L, first i/p info FI_I, TM_X and TM_K are transformation matrices.

**Outputs:** Authorized Integrated Biometric measurement AIB_M

**Step 1:** Declared and then initialized i/p cloud user attribute based parameters along with bi-linear cyclical hash vector. BCH_V [block-bytes/32] ← 0. Initially make bi-linear cyclical hash vector value as NULL.

**Step 2:** Choosing any randomized bi-linear polynomial graph value with private key P_(K)_. It can enhance the overall privacy and security with chaotic arbitrary behavior which is based on logistic model of Bernoulli which leads to generation of strong chaotic randomized practical structure [16]. Logistic plotting is an orthodox frantic plotting exercise whatever the results generated through this model are extremely complicated with chaotic structure. The making of state run follows the below equation

r(b + 1) = β(Ci) (1 + x(Ci))(4)

Here, β deceits from 0 to 1 with randomized time band of bi-linear polynomial dynamic construction. The above-mentioned equation can produce a wide range of chaotic bi-linear cyclic sequence.

B(x) = r(k) × b(x)(5)

Here B(x) is a type of curve taken from the set of bi-linear polynomial curves and r(k) is a randomized factor taken from group Z.

**Step 3:** Calculate

(6) $$µ=\mathrm{LCM}\left(r_{e1}-1,r_{e2}-1\right)\;\mathrm{and}\;Þ=\mathrm{GCD}\left(r_{e1}+1,r_{e2}+1\right),\;n=r_{e1}\times r_{e2}$$

where r_e1_ and r_e2_ are the rudiments taken from bi-linear cyclical set *Z*(m, ∗).

**Step 4:** Opt an arbitrary value which act as co-prime to µ and Þ. Based on *λ* and Þ, calculate r_p1_ and r_p2_

(7) $$r_{p2}=\frac{Ω}{\left(r_{p1}+2\right)}$$

**Step 5:** Select any randomized inputs r_v1_ and r_v2_ which are taken from *Z*(m*^n^*, ∗)

**Step 6:** Determine PM_K1_, PM_K2_, PM_K3_, PM_K4_ as shown below:

PM_K1_ = 1 + r_v3_ × (r_p1_ × r_p2_) (8)

PM_K2_ = pow (PM_K1_, r_v2_) mod (m^2^) (9)

PM_K3_ = S_m1_· pow (PM_k1_, r_v1_^2^) mod (m^2^) (10)

PM_K4_ = S_m1_·PM_k3_·q1· (r_e1_ × r_e2_) mod (m^2^) (11)

**Step 7:** Session key SK_P1_ = {PM_K2_, PM_K3_, PM_K4_, S^m1^, c_g1_·c_g2_, B(m)}.

**Step 8:** Session key SK_P2_ = {m_1_, r_v1_, r_v3_, *α*, r(k)}

**Step 9:**

while (CP_L>Block_Bytes/32)

{

BLK_M ← First 32 bytes of sub block

for all parts of block BLK_M 

{

for initial bit to CS_I

{

opt SK_P1_, SK_P1_ keys with PM_k1_, PM_K2_ as arbitrary transformation box.

PM_k1_ = (*pow*(PM_k2_, *T*), PM_k1_) *mod*(*min*{PM_k1_ · *randomvalues*( )}) *x*1 = PM_k1_ · *T* ∗ PM_k2_ · *scale*(1024) *r_i_* = *BLK*_*M*[*j*] + *s*[*min*(1, *j* − 1)] *r_i_*_+1_ = *min*{*Rv*1, *Rv*2} ⊕ *r_i_* ⊕ *x_i_*

}

*BLK*_*M* ← RIghtShift (*BLK*_*M[j*]) 

*BLK*_*M[j*] ← LeftForward (*BLK*_*M[j*], 10)

if(s+i<CS_I)

{

*BLK*_*M[j*] ← LeftReverse (*BLK*_*M[j*], 6)

*BLK*_*M[j*] ← RightShift (*BLK*_*M[j*], 12)

*BLK*_*M[j*] ← LeftShift (*BLK*_*M[j*], 6)

}

*C* = *c*_0_ + *c*_1_ …… *c_n−r_*

}

### 3.2. Bi-Linear Coupling

Bi-linear coupling produces the multiplicative of any two pairs belonging to a polynomial cyclical set G. Once G is a state of identical elements what we selected from the pair the same pair produces a cyclic polynomial bi-linear relative set. Therefore, the bi-linear coupling gives a wide-range of randomized multiplicative pairs. Let G be a commutative cyclical group with transposed set t, and imagine that g_1_, g_2_, and g_3_ are G-pairs [5]. The combination of each G-bi-linear map gbp: g_1_ × g_2_ → g_3_; i.e., it must follow

gbp (c·g_1_, g_2_) = gbp(g_1_,G·g_2_) = G·cbp(g_1_,g_2_)

gbp (g_11_+ g_12,_ g_2_) = gbp (g_11,_ g_2_) + gbp (g_12,_ g_2_)

gbp (g_1,_ g_21_ + g_22_) = gbp (g_1,_ g_21_) + gbp (g_1,_ g_22_)

For all g ∈ G and whole g_1_, g_11_, g_12_ ∈ G parallel whole g_2_, g_21_, g_22_ ∈ G_2_ which is an optimal pair of G-bilinear map. G_1_⊗_g_ G_2_ → G_3_ and G_1_⊗_G_ G_2_ indicates the multiplicative of G_1_, G_2_. A bilinear curve is also treated as a G-bilinear family of cures iffy and only if Ø: G_3_ → HomoM (G_2_ G_1_); which exactly suits through the definition by representing as Ø (g_3_) (g_2_): = ê(g_3_, g_2_). A G-bilinear family of curves said to be finite and apt if and only if given randomized curve instance Ø is also an isomorph of all G-pairs. A G-bilinear combination of curves are said to be not a degenerative type if and only if ê(g_3_, g_2_) = null to all g_3_ belongs to g_2_ as same as null; similarly, ê is described as not degenerative if the pair (g_3_, g_2_) is also null for all g_2_ belong to g_3_ also said to be null. So, if all above mentioned constraints are satisfactory further the polynomial set of bi-linear curves can be valuated. This practice is efficiently combined with Bernoulli logistic map of family curves of the same type which provide more security in terms of chaotic randomization and enactment [17,23,24,49].

### 3.3. Chaotic Key Making with BQKD

Block chain based QKD uses numerous internetwork communication links, which are a combination of secure quantum link and a general user info link [19,24,36,37,38,39]. The source and destination together get a randomized pair of values on any place of G-bilinear cyclical group by adding cubits which are generated via different polarizers. The planned prototype combined an improved BB-84 authentication decorum which helps to create a randomized key which can access intended cloud users only but not even to cloud admin which avoids MIM bouts [49]. The session wise random key is generated with help of BQKD is supplied to licensed cloud users for the proposed model. The proposed model needs four basic methods, which are QKey_Pdn, I_Setup, CP-En_cipher, and CP-De_cipher.

### 3.4. I_Set_Up Step

Let G be the cyclic bi-linear pair of curves with co-prime order Co and originator O_k_ which must fulfill the G-bilinear principle and compliment degenerative principle so that Ø_1_, Ø_2_ belongs to G_CO_. The public key and master key generated as shown below.

Public-Key(Pb_k_) = {Offered_BQKD(Quantum Key & Consumer attributes) where (g_1_ϵ G_1_(CH_V_[j]), g_co_ ϵ G_2_(CH_V_[j] where G_1_, G_2_ are Integrity metrics (CH_V_,CH_V_[j] ϵ Z^2^_r_, O_k_ = random(g_1_,g_co_)} (12)

Matser_Key(M_k_) = (µ ϵ Pb_k_(g_co_), Þ ϵ BQKD(Private key(CH_V__Att0[1] ^ CH_V__Att1[2] ^ … CH_V__Att_n−1_[n])), Z^2^*, random(µ, Þ)^Ø^_2_} (13)

In this way we can generate Master key (M_k_), BQKD private key (Prk), and public key factors (Pb_k_).

### 3.5. CP-Encipherment Structure

The enciphered procedure gets the cloud consumers unique private info **Up** which as initial i/p through this we can make get final users ciphered text. Next we can encipher the cloud consumers unique private info **Up** which can be utilized to get control over the access policy **Ap**. Base from the starting point vertex **Sv**, our approach selects a randomized number **Rn** from mod (abs (random integers, Z^2^) and institutes **q(Rk,0) = Rn** which understand that the middle level vertices, **Mv**, are arranged like (In,0) = Q (Sv(Rn,index_value)). Assume **Lv** to be the set of leaf vertices at the control access structure/policy; from this ciphered text can be generated over the existing access structure tree of policies **Ast** as shown below:

Ciphered Text(Ct) = {Ct^0^ = Pt. rand(R1,Rn)^Ø^_1_^.rv^, Pt, Ct^1^ = b^rv^ ∀ In ϵI_N: C_y_ = R1^qkd(in,1)^,
Cy^1^_in_ = CHv(A(In)^qkd(in,n)^ (14)

Confirming overall homomorphism principles to mien original text message enciphering:

Homomorphism based encipher, decipher uses Ω(*r*0), Ω(*r*1) as initial values.

Ω(*r*0) = CPEncD (*j*_1_): = CPEncD(*j*_1_) = (*j*_1_ + *γ*µ ∗ Þ) mod Me Ω(*r*1) = CPEncD(*j*1): = CPEncD(*j*2) = (*j*_2_ + µ ∗ Þ) mod Me whereas Me = *α* ∗ *β*; *CPEncD*(*j*_1_ + *j*_2_): = CPEnc(Ω(*r*0)) + CPEnc(Ω(*r*1));

CPMEncD(*j*.*j*_2_): = CPMEncD(Ω(*r*0)).CPMEncD(Ω(*r*1)): = (*j*_1_ + µ ∗ Þ) mod Me + (*j*_2_ + µ ∗ Þ)mod Me.

### 3.6. QK_Pdn Step

The session wise key production process can generate Private key (P_rk_) by using cloud consumers’ set of personal parameters (S_pp_). The QK_Pdn process ingests cloud consumers parameter set S_pp_ act as initial i/p for BQKD (Open key), and the formed o/p is the secret key. This process opts for a couple of arbitrary numbers, A1 and A2; for combination of each cloud consumer parameter set S_pp_. These random values are part of selected co-factor of BQKD (shared key) which belongs to Z^n^_p_.

Private_key(P_rk_) = {Qk(0) = kv^(^^Ø^^1^^+A2)/^^Ø2^, QK(f) = kv^randf*Chv(f).A2^ } (15)

### 3.7. CPDecipherment Process

In this process a private key P_rk_, cloud consumers set personal parameters S_pp_, ciphered text Ct along with access policy tree structure (Ast) and public-key (Pb_K_) as i/p. The deciphering procedure is implemented iteratively. Verification of homomorphism property for user information decipherment is as follows: let be P_rk_.S_pp_(*CPEnc*(*j*_1_ + *j*_2_), *MCPEnc*(*j*_1_.*j*_2_). S_pp_(*Ct*(*j*)_1_, *K*_1,i_). S_pp_(T*K*_1,j_). S_pp_(*Ct*(*k*)_3_,*K*_1,f_) to get the created cloud consumer control structure.

CPEnc(*j*_1_ + *j*_2_) = CPEnc (Ω(*r*0) + Ω(*r*1)) = CPEnc(Ω(*r*0)) + CPEnc(Ω(*r*0)); : = (Ω(*r*0) + µ ∗ Þ)mod Me + (Ω(*r*1) + µ ∗ Þ)mod Me (16)

CPEnc(*j*_1_.*j*_2_) = CPEnc(Ω(*r*0) ∗ Ω(*r*1)=): = CPEnc(Ω(*r*0)).Enc(Ω(*r*1));
: = (Ω(*r*0) + µ ∗ Þ)mod Me + (Ω(*r*1) + µ ∗ Þ)mod Me (17)

CPDec(*EncD*(*j*_1_ + *j*_2_)): = (CPEncD(Ω(*r*0) + Ω(*r*1)) mod *β*
: = ((Ω(*r*0) + µ ∗ Þ)mod Me + (Ω(*r*1) + µ ∗ Þ)mod Me) mod *β*: = *j*_1_ + *j*_2_ (18)

CPDec(EncD(*j*_1_.*j*_2_)): = (CPEncD(Ω(*r*0) ∗ Ω(*r*1)): = CPEncD(Ω(*r*0)).CPEnc(Ω(*r*1))mod*β*
: = ((*ω*(*i*)_0_ + µ ∗ Þ)mod Me + (*ω*(*i*)_0_′ + µ ∗ Þ)mod Me) mod *β*: = *i*_1_.*i*_2_ (19)

## 4. Experimental Results

The experimentations can be situated and executed over simple storage service (AWS-S3) with help after cloud consumer device details: processor i7 with speed of 2.30 GHz, 16 GB primary memory, 64-bit OS. This outline requires the usage of standard inbuilt packages and interfaces like EC2, Java, Net beans, and Eclipse.

### Cloud Platform Base

AWS cloud services are utilized in the projected secure block chain based cloud environment to act out the projected model on users’ confidential clinical and medical data. We used Amazon Elastic Compute Cloud and S3 buckets for generation of investigational outcomes to achieve effective public cloud security me integrating our proposed algorithms to the model. Elastic Compute Cloud offers upfront and tranquil cloud centric large scale calculations for all types of consumers. Elastic Compute Cloud cases are setup on Virtual Private Cloud. Using this cloud, consumers can do their trails in all possible cases with low cost for reliable services. For experimentation and analysis of Elastic Compute Cloud cases AWS, compromises an Internet based support, i.e., Cloud_Watch [2,6,7,8,9,13,14,15,16,19]. Cloud_Watch supervises resource reservation administration dynamically. Elastic Compute Cloud enables cloud consumers to opt for various functional objects, upgrade existing plans, 24/7 monitoring and management, along with emulation of executing capability in prior.

**Figure 9:**
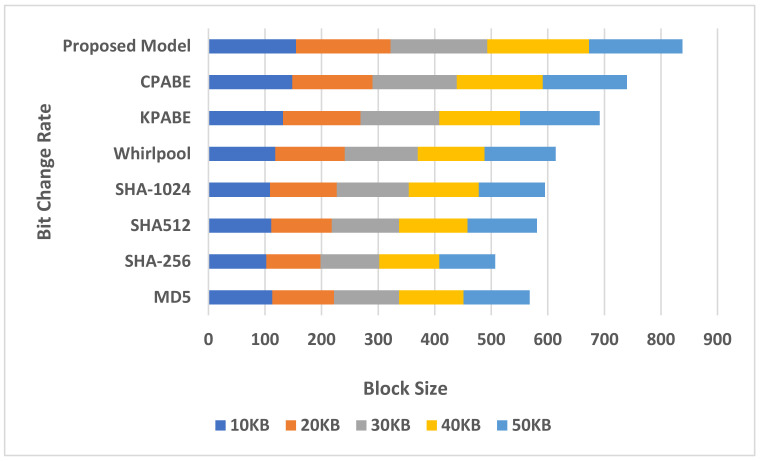
Computational time band for chaotic key production.

Figure 9 shows a comparison between the proposed model and the existing models with regard to changing the hash bit change. The tabletop showed the proposed method has a higher value than the standard models.

**Figure 10:**
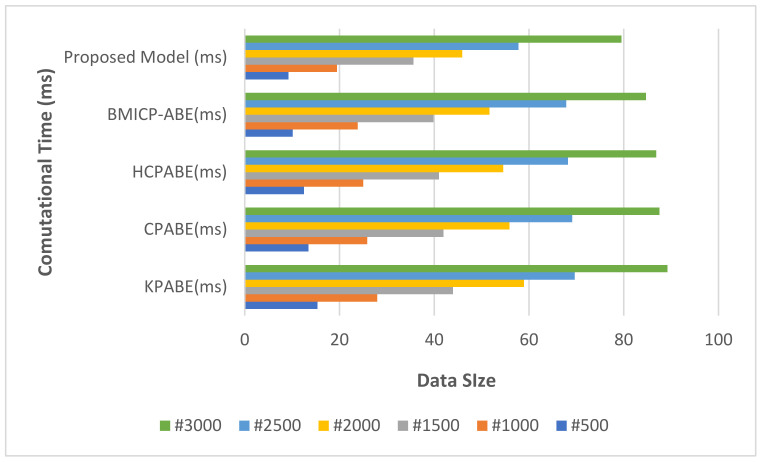
Comparative Analysis of Average Computational Time.

The projected method is compared with the past models; runtime calculations are illustrated in Figure 10. In the experimental trial, the average processing time is determined by varying the data sizes. The figure shows that the proposed models have good cloud safety computational efficiency.

**Figure 11:**
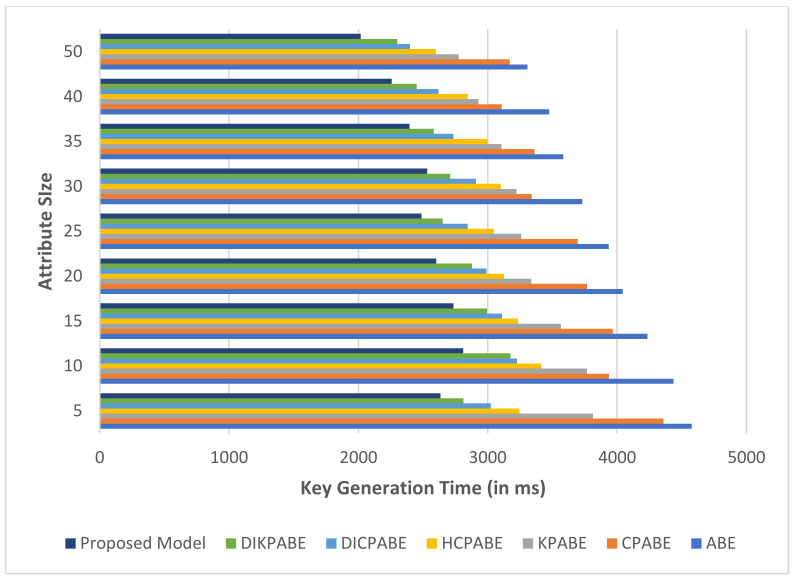
Comparative analysis of chaotic key creation time band and whole amount of cloud consumers’ attributes.

Figure 11 represents the computation time wanted on average for the projected technique with the past approaches with respect to the chaotic key construction process. In this projected technique, the chaotic dynamic key production system complete computation time is far lower than those of the available models.

Table 1 typifies the efficiency of the projected model with respect to past approaches validation process by means of bit change and dynamic randomized hash calculation. According to Table 2 above, the present dynamically randomized reliable approach is far better than the existing approaches for the production of chaotic hash values; ms stands for message size and kl signifies key length.

Table 2 presents a proportional investigation of the acclaimed parameters with the traditional models. Table 2 shows the projected method has separate value added points like static key, a large volume of cloud consumers’ personal information, and randomized chaotic session wise dynamic key creation process. The projected technique has one more asset: that it has extremely chaotic randomized flexible key sizes along with the fact that it takes far less communiqué network overhead compared to the related traditional methods.

## 5. Conclusions and Future Scope

The present approach provides a novel and enhanced bilinear QCPABE chaotic randomized key generation over the secure block chain cloud environment based user’s sensitive data. The proposed approach uses a group of bilinear polynomial curves by means of extensively randomized complex chaotic utility. Conventional ABE approaches fail to handle the cloud consumer’s large volumes of sensitive information and most existing methods are independent on integrity parameter due to lack of computational resources with less computational overhead. Our approach addresses all the traditional approaches issues and problems. Our proposed approach was implemented and successfully functional over the cloud consumers’ large volume sets of sensitive data which are in the format of structured, semi structured, and non-structured. Our proposed approach can secure the cloud consumers’ personal attributes by applying bilinear polynomial chaotic randomized map function designed for key setup, encipher, and decipher mention. The experimental replication results proved that our approach has the finest accuracy and rightness with respect to time required for dynamic key generation, encipher and decipherment process, and needed far less memory and computational overhead over the traditional approaches. Compared to existing models (CPABE, CQ-CPABE, KPABE, and QCP-ABE types), the real-time simulation results demonstrate that the stated standard is more precise than 90% in terms of bit change and more precise than 95% in terms of dynamic key generation, encipherment, and decipherment time. In the future this work may be protracted to advance the efficiency of the encryption and decryption process for the multi-document file formats using deep learning framework without loss of quality and resolution of the users various data representations over the cloud.

## Figures and Tables

**Table 1:** Conventional Hash Technique-based proposed model efficacy.

Cloud Clinical Data-Set	MD5	QCP ABE	SHA-1024	Whirlpool	Proposed Model
Efficiency	O(ms^2kl)	O(ms log(kl))	O(ms kl log(n))	O(ms log(ms kl))	O(ms log(kl/2))

**Table 2:** Summary of comparisons of state-of-the-art literature with proposed method.

Properties	MD5	SHA-256	Whirlpool	SHA-512	QCP ABE	SHA-1024	Proposed Model
Key length	Static	Static	Static	Static	variable	Static	Randomized
Dynamic key	Nope	Nope	Nope	Nope	Sure	Nope	Sure
Massive data	Nope	Nope	Nope	Nope	Sure	Nope	Sure
Transmission cost	More	More	More	More	Slightly Less	More	Very Less
Static key	Sure	Sure	Sure	Sure	Sure	Sure	Sure

## Data Availability

Data are available on request due to restrictions, e.g., privacy or ethical reasons. The data presented in this study are available on request from the corresponding author. The data are not publicly available due to privacy reasons.
